# Climates and associated factors for evidence-based practice implementation among nurses: a cross-sectional study

**DOI:** 10.1186/s12912-023-01694-y

**Published:** 2024-01-22

**Authors:** Xinyue Zhang, Mengting Peng, Mei He, Meijie Du, Mengyao Jiang, Mengying Cui, Yue Cai, Qi Yan, Ying Wang

**Affiliations:** 1 Nursing Department, Tongji Hospital, Tongji Medical College, Huazhong University of Science and Technology, 430030 Wuhan, Hubei China; 2 School of Nursing, Tongji Medical College, Huazhong University of Science and Technology, 430030 Wuhan, Hubei China

**Keywords:** Evidence-based practice, Organizational implementation, Implementation climate, Nursing

## Abstract

**Background:**

The organizational climate that fosters and supports the implementation of evidence is a key factor influencing the effective implementation of evidence-based practice (EBP). Nurses, being the largest group of medical staff, play a crucial role in EBP implementation. The perception of the climate for EBP implementation among nurses in their organizations is unknown, especially among Chinese nurses.

**Aims:**

To clarify the implementation climate of EBP among Chinese nurses and identify the factors associated with the implementation and development of evidence-based nursing practices.

**Methods:**

This study employed a descriptive cross-sectional study design. From March 2023 to April 2023, a sample of nurses (n = 1260) from two Tertiary care hospitals in central China were selected and surveyed by self-designed social-demographic questionnaire and Implementation Climate Scale. Multiple linear stepwise regression analysis was conducted to determine the predictors of implementation climate.

**Results:**

The nurses achieved a mean ICS score of 59.10 ± 11.22, with mean scores exceeding 3 points for each sub-dimension and item. In the results of multiple linear regression, income satisfaction, implementation of evidence-based nursing practice project(s) within the unit, experience of evidence-based nursing practice during working life, and specific training or courses in evidence-based nursing practice were predictors of ICS. Of these, income satisfaction was the most significant predictor. These factors could explain 17.5% of the total variance in implementation climate.

**Conclusion:**

The climate for implementing EBP in Chinese nursing organizations was relatively strong. Nursing managers can enhance the climate for implementing EBP in their organizations by actively improving salary and enhancing EBP-related trainings and practices.

**Relevance to clinical practice:**

Understanding nurses’ perceptions of the EBP implementation climate in their organizations can help to identify specific barriers and facilitators to the development of EBP and facilitate its successful implementation.

**Patient or public contribution:**

Clinical nurses were involved in data collection and completed the questionnaires on EBP implementation climate.

**Supplementary Information:**

The online version contains supplementary material available at 10.1186/s12912-023-01694-y.

## What does this paper contribute to the wider global clinical community?

The number of studies on the organization and implementation climate of evidence-based nursing practice is limited, especially in China.

According to the results of this study, nurses exhibited a positive perception and recognition of the organized EBP implementation climate.

Income satisfaction, implementation of evidence-based nursing practice project(s) within the unit, experience of evidence-based nursing practice during the working life and specific training or courses undertaken in evidence-based nursing practice were factors of nurses’ perception of implementation climate.

Organizational managers should actively provide comprehensive resource support to enhance the implementation climate of evidence-based nursing practice.

**Supplementary Information:**

The online version contains supplementary material available at 10.1186/s12912-023-01694-y.

## Introduction

As a long-standing problem-solving and extensively employed scientific approach, EBP integrates research evidence, clinical expertise, and patients’ preferences and values to establish evidence as the foundation for clinical nursing decision-making [1]. Globally, nursing is widely acknowledged as a pivotal element in the healthcare system, with nurses delivering the majority of direct patient care. The incorporation of EBP in nursing has demonstrated improvements in healthcare quality, heightened reliability, and enhanced patient outcomes [2–4].

However, nurses still encounter obstacles in utilizing and successfully implementing evidence in clinical practice. Various factors can hinder the integration of evidence into clinical practice. These obstacles include individual, social, and organizational factors, as well as patient-related factors, and those specific to the innovation itself [5]. Individual factors, such as skill level, attitude, and motivation, can all influence a healthcare provider’s capacity to integrate new evidence into their practice. In addition, social and organizational factors, such as leadership, available resources, and institutional culture, can significantly influence the adoption of EBPs. Patients’ knowledge and compliance with treatment plans can also influence the adoption of evidence. Lastly, the innovation itself may pose specific barriers, such as cost, feasibility, and accessibility, which can all influence the integration of a new treatment or intervention into clinical practice.

Among these factors, organizational factors that promote and support evidence implementation are key determinants affecting the effective implementation of EBP. When EBP is delivered in a context of caring and a culture, supported by an ecosystem or environment, the best clinical decisions are made, resulting in positive patient outcomes [4]. In the field of EBP, the implementation climate refers to the collective awareness and acknowledgment within an organization of the significance and value of utilizing the best available evidence. Assessing the implementation climate can provide insight into the attitudes, expectations, and perceptions of the organization’s members regarding the adoption, implementation, and utilization of EBPs. This measurement can help determine the level of support, rewards and assistance that members expect or need to engage effectively with EBP. Understanding nurses’ perceptions of the implementation climate of EBP in their organization is crucial for identifying the specific barriers and facilitators impacting the implementation process. This information can be used to promote the effective and smooth development of EBP within the organization. Identifying the factors that hinder or support the adoption and utilization of EBP can help develop appropriate strategies and interventions to overcome obstacles and enhance the implementation climate.

## Background

In organizational studies, the concept of climate holds significance with a long historical background. A theoretical model proposed in 1996 suggested that organizations can develop a positive implementation climate by employing policies and practices that encourage members to utilize innovative means, motives, and opportunities [6]. This underscores the importance of fostering an organizational culture that supports and incentivizes innovation to facilitate progress and achievement. The model proposes that the implementation climate, which refers the extent to which organizational members perceive encouragement, facilitation and rewards for innovative use, is positively correlated with implementation effectiveness. In other words, organizations that promote a culture supporting innovation and provide members with the necessary resources and support for effective use are more likely to successfully implement innovative practices [6, 7].

Organizational climate is employed to facilitate a suitable pre-assessment of organizational context and the formulation of strategies to expedite effective implementation. This is defined as “the shared meaning organizational members attach to the events, policies, practices, and procedures they experience and the behaviors they see being rewarded, supported, and expected” [8, 9]. Implementation climate differs from constructs like organizational climate, culture, or context in two crucial respects: firstly, it has a strategic focus (implementation), and secondly, it is innovation-specific. In other words, organizational climate is a broader concept that encompasses the overall atmosphere and shared perceptions within an organization. In the context of organizational climate, implementation climate places more emphasis on implementation and innovation. Simultaneously, implementation climate signifies a high degree of within-group agreement in climate perceptions [7]. Therefore, recognizing the attitudes of organizational members towards implementing climate and intensity changes is crucial for changing their behavior, and can help facilitate successful realization of the implementation and expected innovation. When it comes to EBP implementation, an effective “implementation climate” refers to how employees perceive the organization’s support and expectation regarding the adoption, implementation, and utilization of such innovation [7, 10, 11]. EBP implementation climate is defined as employees’ shared perceptions of the importance of EBP implementation within the organization [11], emphasizing the creation of fertile organizational context for putting EBPs into practice [8].

The Implementation Climate Scale (ICS) was developed to assess the organizational context for EBP implementation, building upon the progress and growth of strategic climate [8]. Currently, it has been used in substance use disorder treatment organizations [12], child welfare organizations [13] acute care [14], and others to assess the implementation climate and its validity. Limited studies have been conducted in the field of nursing, and to the best of our knowledge, there have been no reports in China.

Certain factors related to EBP implementation among nurses have garnered increased attention. Despite the attention given to factors such as knowledge, attitudes [15], leadership [14], and EBP utilization [3] in previous studies, the understanding of the implementation climate and its related influencing factors regarding EBP among nurses is still limited. Given the relatively recent inception of EBP in nursing, it is crucial to enhance the understanding of the organizational and implementation climate, along with the existing obstacles, to facilitate improvement. Therefore, this cross-sectional study is designed to investigate the implementation climate and associated factors among nurses in China.

## Methods

### Design and participants

This study utilized a descriptive cross-sectional study design. Clinical nurses were selected as research subjects using a mixed sampling method involving both convenience sampling and stratified sampling techniques. Nurses from two Tertiary care hospitals in central China were specifically chosen for the study. Both hospitals had approximately 4,000 nursing staff. The survey was conducted at a ratio of 3:1 between our hospital and the other hospital. The inclusion criteria for nurses in this study were as follows: (1) being registered nurses in China, (2) having a minimum working experience of one year, (3) being at least 18 years old, and (4) providing informed consent and willingly participating in the study. Intern nurses and nurses on maternity leave, sick leave, or absent for any other reasons during the investigation were excluded.

The sample size was determined using G*Power 3.1.9.4 software, with the linear multiple regression algorithm selected as the statistical analysis method. Considering an effect size of 0.15, a significance level of 0.05, a power of 0.8, and 12 predictors, the minimum required sample size was determined to be 127. Accounting for a dropout rate of 20%, the total estimated sample size needed for the study was no less than 159 participants.

### Measures

The questionnaire mainly covered two aspects of information. The first part was based on the relevant research results of existing studies on the topics of “nurses” and “EBP” in existing studies, and the questionnaire was self-designed by the researchers, including the participants’ social-demographic characteristics and the factors related to the study, including participants’ gender, age, education level, position, professional title, years of work experience, involvement in night shift work per week, income satisfaction, implementation of evidence-based nursing practice project(s) within the unit, any experience of evidence-based nursing practice during the working life, and specific training or courses undertaken in evidence-based nursing practice.

The second aspect of the questionnaire included the Chinese version of the ICS, initially developed by Ehrhart et al. [8] and translated into Chinese by Bai Wenhui et al. [16]. The scale consisted of 18 items, categorized into 6 sub-dimensions: focus on EBP, educational support for EBP, recognition for EBP, rewards for EBP, selection for EBP and selection for openness. Each item was assessed using a 5-point Likert scale with response options ranging from 0, indicating “not at all,” to 4, indicating “very great extent”. The total score on the scale ranged from 0 to 72, with a higher score indicating a greater level of implementation climate for EBP. The original scale demonstrated reliability with Cronbach’s α for the 6 dimensions ranging from 0.81 to 0.98, and in this study, Cronbach’s α was 0.90 to 0.96.

### Data collection procedure and ethical consideration

To ensure representative samples and convenient data collection, we used a stratified sampling method. Clinical nurses were categorized into 9 major departments (surgery, internal medicine, pediatrics, emergency, etc.), and the survey sample size was determined based on each department’s personnel proportion. Convenience sampling was then used within each department during the actual sampling. The specific data collection process is outlined as follows: from March 2023 to April 2023, after approval from the nursing department, the questionnaire was sent to department head nurses, who then shared the electronic questionnaire link with clinical nurses meeting the inclusion criteria. The researchers distributed the questionnaire with clear instructions, explaining the survey’ s purpose and significance. Additional verbal clarification was given by the head nurse when distributing the questionnaire. Furthermore, the researcher’ s contact number was provided in the questionnaire for participants to seek clarification at any time. Information on questionnaire principles and confidentiality was shared, with all details communicated after obtaining informed consent. Participants had to answer all questions before submitting. A total of 1,278 questionnaires were collected. After excluding questionnaires with incomplete data, 1,260 valid questionnaires were obtained, resulting in a valid return rate of 98.59%. The study underwent review by the Medical Ethics Committee of the hospital (No.TJ-IRB20221114). All materials and information related to the specific questionnaire were accessible only to the researchers.

### Data analysis

Data integration and statistical analysis were performed using SPSS 26.0 software. Before analysis, the dataset underwent examined for missing values, outliers, and adherence to normality assumptions. The Student t test (for dichotomous variables), one-way ANOVA, Welch F-test, and Least-Significant Difference (for polytomous variables) were conducted for univariate analysis. Multiple linear regression analysis was performed to separately examine the related factors of the scale and each dimension. To address the potential issue of multicollinearity, stepwise regression analysis was employed in this study. The significance levels for variable entry (*α*_in_) and removal (*α*_out_) were set at 0.05 and 0.10, respectively. Multicollinearity was assessed by examining tolerance and the variance inflation factor (VIF). Tolerance values below 0.10 or VIF values above 10 were indicative of multicollinearity. A *p*-value of less than 0.05 (two-tailed) was considered statistically significant for interpreting the results.

## Results

### Participants’ characteristics

The general characteristics of the participants were presented in Table 1. Overall, the participants had an average age of 32.49 ± 6.80 years, and 97.30% of them were female. The majority of the participates did not hold a nursing position (93.89%). 53.25% of the nurses held senior positions, and 27.70% were supervisors. Regarding educational level, 94.68% held an undergraduate degree. The nurses had varied work experience, with 82.14% having experience ranging from 1 to 15 years. Furthermore, 80.63% of the nurses reported having weekly night shift work, while only 4.44% expressed dissatisfaction with their current income. In terms of EBP experience, 73.73% of the nurses reported that their units had implemented evidence-based nursing practice project(s). Moreover, 66.03% of the nurses had prior experience with evidence-based nursing practice during their working life, and 70.32% had received specific training or courses in evidence-based nursing practice.

**Table 1:** Examination of the nurses’ mean ICS scores based on different characteristics (n = 1260)

Variables	*n* (%)	Mean ± *SD*	Test value	*p*
Age				
20–25 years	194 (15.40)	55.90 ± 10.01	*F* = 7.860^a^	<0.001^**^
26–30 years	351 (27.86)	58.27 ± 11.48		
31–35 years	391 (31.03)	60.21 ± 11.51		
36–40 years	175 (13.89)	60.18 ± 10.58		
>40 years	149 (11.83)	61.04 ± 11.16		
Gender				
Female	1226 (97.30)	59.17 ± 11.23	*t* = 1.920^b^	0.166
Male	34 (2.70)	56.47 ± 10.60		
Position				
Yes	77 (6.11)	60.96 ± 10.77	*t* = 2.261^b^	0.133
No	1183 (93.89)	58.98 ± 11.24		
Professional title				
Primary nurse	218 (17.30)	56.75 ± 10.72	*F* = 4.867 ^a^	0.003^*^
Senior nurse	671 (53.25)	59.18 ± 11.13		
Supervisor nurse	349 (27.70)	60.36 ± 11.59		
Co-chief nurse and above	22 (1.75)	59.95 ± 9.79		
Education level				
Junior college	9 (0.71)	67.56 ± 8.88	*F* = 2.650^c^	0.071
Undergraduate	1193 (94.68)	59.01 ± 11.21		
Postgraduate and above	58 (4.60)	59.57 ± 11.44		
Years of work experience				
1–5 years	272 (21.59)	55.72 ± 10.24	*F* = 10.383^a^	<0.001^**^
5–10 years	420 (33.33)	59.19 ± 11.31		
10–15 years	343 (27.22)	60.87 ± 11.29		
15–20 years	99 (7.86)	59.29 ± 11.11		
＞20 years	126 (10.00)	61.09 ± 11.36		
Involvement in night shift work per week				
Yes	1016 (80.63)	59.01 ± 11.26	*t* = 0.304^b^	0.581
No	244 (19.37)	59.45 ± 11.06		
Income satisfaction				
Not satisfied	56 (4.44)	53.54 ± 12.56	*F* = 81.219^a^	<0.001^**^
Mildly satisfied	341 (27.06)	55.09 ± 10.82		
Moderately satisfied	715 (56.75)	59.65 ± 10.79		
Extremely satisfied	148 (11.75)	67.79 ± 7.50		
Implementation of evidence-based nursing practice project(s) within the unit				
Yes	929 (73.73)	61.20 ± 10.27	*F* = 70.314^c^	<0.001^**^
No	122 (9.68)	54.37 ± 12.21		
Not sure	209 (16.59)	52.30 ± 11.31		
Experience of evidence-based nursing practice during the working life				
Yes	832 (66.03)	61.13 ± 10.62	*F* = 82.433^a^	<0.001^**^
No	428 (33.97)	55.15 ± 11.31		
Specific training or courses undertaken in evidence-based nursing practice				
Yes	886 (70.32)	60.90 ± 10.55	*F* = 75.625^a^	<0.001^**^
No	374 (29.68)	54.84 ± 11.61		

^a^ Welch F-test

^b^ Student t test

^c^ One-way ANOVA test

^*^p < 0.01; ^**^p < 0.001

### Nurses’ implementation climate

The mean ICS score for nurses was 59.10 ± 11.22. Mean scores for each sub-dimension were as follows: 9.90 ± 1.99 for “focus on EBP”, 9.89 ± 1.98 for “educational support for EBP”, 9.80 ± 2.00 for “recognition for EBP”, 9.71 ± 2.08 for “rewards for EBP”, 9.85 ± 1.98 for “selection for EBP” and 9.94 ± 1.93 for “selection for openness” (Table 2). The scores across dimensions were relatively balanced, indicating that clinical nurses had an overall positive perception of the implementation climate. However, the mean score for dimension “rewards for EBP” was the lowest, suggesting that clinical nurses had a less favorable perception of rewards in the implementation of EBP. A detailed examination of individual ICS items (Table 3) revealed that the item ‘people in this team/agency think that the implementation of EBPs is important’ had the highest mean score of 3.34 ± 0.66, while the item ‘this team/agency provides the ability to accumulate compensated time for the use of EBPs’ scored the lowest with the mean score of 3.21 ± 0.75.

**Table 2:** Descriptive statistics for the nurses’ ICS total and sub-dimensions scores

Variables (Possible score range)	Range	Mean ± *SD*
ICS total score (0–72)	18–72	59.10 ± 11.22
ICS sub-dimensions		
Focus on EBP (0–12)	0–12	9.90 ± 1.99
Educational support for EBP (0–12)	0–12	9.89 ± 1.98
Recognition for EBP (0–12)	2–12	9.80 ± 2.00
Rewards for EBP (0–12)	0–12	9.71 ± 2.08
Selection for EBP (0–12)	3–12	9.85 ± 1.98
Selection for openness (0–12)	3–12	9.94 ± 1.93

**Table 3:** Descriptive statistics for the nurses’ ICS item scores

Items	Mean ± *SD*
1. One of this team/agency’s main goals is to use evidence-based practices effectively	3.27 ± 0.72
2. People in this team/agency think that the implementation of evidence-based practices is important	3.34 ± 0.66
3. Using evidence-based practices is a top priority in this team/agency	3.29 ± 0.71
4. This team/agency provides conferences, workshops, or seminars focusing on evidence-based practices	3.28 ± 0.72
5. This team/agency provides evidence-based practice trainings or in-services	3.31 ± 0.68
6. This team/agency provides evidence-based practice training materials, journals, etc.	3.30 ± 0.68
7. Clinicians in this team/agency who use evidence-based practices are seen as clinical experts	3.26 ± 0.71
8. Clinicians who use evidence-based practices are held in high esteem in this team/agency	3.28 ± 0.69
9. Clinicians in this team/agency who use evidence-based practices are more likely to be promoted	3.26 ± 0.69
10. This team/agency provides financial incentives for the use of evidence-based practices	3.22 ± 0.74
11. The better you are at using evidence-based practices, the more likely you are to get a bonus or a raise	3.28 ± 0.69
12. This team/agency provides the ability to accumulate compensated time for the use of evidence-based practices.	3.21 ± 0.75
13. This team/agency selects staff who have previously used evidence-based practice	3.28 ± 0.68
14. This team/agency selects staff who have had formal education supporting evidence-based practice	3.29 ± 0.68
15. This team/agency selects staff who value evidence-based practice	3.29 ± 0.67
16. This team/agency selects staff who are adaptable	3.32 ± 0.65
17. This team/agency selects staff who are flexible	3.31 ± 0.66
18. This team/agency selects staff open to new types of interventions	3.32 ± 0.66

### ICS scores of nurses with different characteristics

Statistical analyses, including the Student t test, Welch F-test, One-way ANOVA and Least-Significant Difference, were conducted to explore various factors influencing ICS scores among nurses (Table 1). The results suggested that age, professional title, years of work experience, income satisfaction, implementation of evidence-based nursing practice project(s) within the unit, experience of evidence-based nursing practice during working life, and specific training or courses in evidence-based nursing practice were statistically significantly associated with ICS (*p* < 0.05). The details were as follows.

Significant differences in ICS scores were observed among nurses of different age groups (*p* < 0.05). Specifically, nurses aged 20–25 had significantly lower ICS scores compared to other age groups (*p* < 0.05), while no significant differences were found among nurses aged 31 years or older (*p* > 0.05). Nurses with less than 5 years of experience had significantly lower ICS scores than those with more than 5 years of experience (*p* < 0.05). Additionally, nurses with 5–10 years of experience had significantly lower ICS scores than those with 10–15 years of experience. Significant differences in ICS scores were also noted among nurses with different professional titles (*p* < 0.05), where the mean ICS scores of primary nurses were significantly lower than those of senior nurses and supervisor nurses (*p* < 0.05). The results also showed that nurses with higher income satisfaction had higher ICS scores (*p* < 0.05). However, no significant differences in ICS scores were found based on gender, position, educational level, or involvement in night shift work per week (*p* > 0.05). In terms of EBP-related factors, nurses working in units with implemented evidence-based nursing practice projects, having prior experience in evidence-based nursing practice, and receiving specific training or courses in evidence-based nursing practice exhibited higher ICS scores compared to their counterparts (*p* < 0.05).

### Multiple linear regression of factors influencing implementation climate

Utilizing variables that exhibited statistical significance in the univariate analysis, a multiple linear stepwise regression analysis was performed to establish the optimal model (*α*_in_ = 0.05, *α*_out_ = 0.10). The results (Table 4) indicated that income satisfaction, implementation of evidence-based nursing practice project(s) within the unit, experience of evidence-based nursing practice during the working life, and specific training or courses in evidence-based nursing practice were predictors of ICS (R^2^ = 0.177, adjusted R^2^ = 0.175; *F* = 67.603, *p* < 0.001).

This study also conducted a detailed analysis of the scores in different dimensions of ICS based on various characteristics and developed multiple linear regression models for each dimension. The results were consistent with the overall score; therefore, the detailed analysis of individual dimensions is not reported in this paper.

**Table 4:** Multiple linear regression analysis results regarding the prediction of ICS scores

Predictor	*B*	*SE*	*β*	*t*	*p*	Model statistics
Constant	57.306	1.706	-	33.598	<0.001^***^	R^2^ = 0.177, adjusted R^2^ = 0.175; *F* = 67.603, *p* < 0.001^***^
Income satisfaction	3.917	0.419	0.249	9.338	<0.001^***^	
Implementation of evidence-based nursing practice project(s) within the unit	-2.606	0.453	-0.176	-5.748	<0.001^***^	
Experience of evidence-based nursing practice during the working life	-1.579	0.758	-0.067	-2.084	0.037^*^	
Specific training or courses undertaken in evidence-based nursing practice	-2.444	0.731	-0.100	-3.345	0.001^**^	

*Note*: B, unstandardized beta coefficients; SE, standard error; β, standardized beta coefficients

^*^p < 0.05; ^**^p < 0.01; ^***^p < 0.001

## Discussion

Implementation climate refers to the organizational factors that influence the successful adoption, implementation, and sustainability of EBPs. An effective implementation climate comprises the attitudes, beliefs, and behaviors of employees concerning the adoption and utilization of EBPs. In this study, nurses achieved a mean score of 59.10 ± 11.22 on the ICS, signifying a relatively elevated level of implementation climate. Furthermore, the mean score for each item on the ICS among clinical nurses surpassed 3 points, presenting a higher outcome in contrast to prior studies utilizing the ICS questionnaire for investigation [12, 14]. These findings imply a positive perception and favorable organizational factors conducive to the adoption and implementation of EBPs among the nurses. Inconsistent findings across different studies may be attributed to various factors, including variations in sample characteristics. In this study, nurses were selected from two Tertiary care hospitals, emphasizing a focus on EBPs. This specific sample characteristic could contribute to the higher scores and more positive attitudes towards the observed implementation climate in this study. The incorporation of EBPs within the hospitals could establish a supportive environment and organizational culture fostering the adoption and utilization of EBPs among the nurses. This circumstance might result in higher implementation climate scores compared to studies conducted in environments with a lesser emphasis on EBPs. Therefore, the unique context and sample characteristics of the study participants in this investigation may explain the discrepancies in findings compared to prior studies. Considering the limited availability of research results on ICS scores, conducting multiple comparisons becomes challenging. Indeed, higher scores indicate a more favorable rating of the organizations’ implementation climate [17]. From the present research findings of this study, it can be inferred that the participating nurses exhibit a strong sense of climate and organizational identity when it comes to evidence-based nursing practice.

The results of this study revealed that the sub-dimension ‘selection for openness’ obtained the highest mean score among the ICS sub-dimensions. This suggests that managers in the surveyed hospitals maintained a positive and fair attitude towards selecting evidence-based practitioners. Building upon these findings, future efforts can concentrate on reinforcing systematic training programs and implementing standardized multi-dimensional assessment systems, aiming to enhance the selection of practitioners. Conversely, the mean score in sub-dimension ‘rewards for EBP’ was the lowest. This implies that a clear incentive policy for evidence-based nursing practice might be lacking in the hospital, resulting in insufficient policy support and resource allocation in the performance management of nurses and related training subsidies. The absence of sufficient incentives could potentially hinder the promotion of clinical practice reform and the adoption of EBPs. To address this issue, nursing managers should consider formulating reasonable incentive policies and ensuring resource guarantees. One effective approach could involve integrating the content of evidence-based nursing practice into the system of performance evaluation, rewards, and professional development. Through this, nurses would be encouraged and motivated to engage in evidence-based nursing practice as part of their professional development and practice behavior. Regarding the evaluation results of ICS items, the items received positive scores from most respondents. However, some variations in certain items can still be identified. Specifically, the item ‘this team/agency provides the ability to accumulate compensated time for the use of EBPs’ scored the lowest. This aligns with the lower score observed in the aforementioned dimension ‘rewards for EBP’, further illustrating the importance of organizing systematic evidence-based nursing training and ensuring relevant resource guarantees to foster climate for the implementation of EBP.

The findings indicated that income satisfaction emerged as an important factor influencing nurses’ perception of the EBP implementation climate. Nurses who reported heightened satisfaction with their current income also tended to perceive a more positive sense for EBP implementation climate. The association between income satisfaction and EBP perception can be explained as follows: when nurses are content with their income, they may experience an enhanced sense of job security and financial well-being. In turn, this can positively influence their overall job satisfaction and engagement in their professional roles, including their willingness to embrace EBPs. The finding also indirectly reflected the lowest score of ‘rewards for EBP’ in the results and the lack of a systematic and reasonable financial incentive mechanism for hospitals. Similarly, the result aligns with the challenges and obstacles mentioned in the analysis of evidence-based nursing implementation in Mainland China, indicating that most barriers to evidence-based nursing practice originate from a system level, including funding from clinical institutes and managerial support [18]. Implementation of evidence-based nursing practice project(s) within the unit and the experience of evidence-based nursing practice during the working life were also independent influencing factors of EBP implementation climate. Existing research showed that evidence-based nursing practice is a crucial method to enhance nurses’ evidence-based ability [18, 19]. Through evidence-based nursing practice, the acquired knowledge is transformed into EBP. This ensures that evidence-based nursing is not only at the theoretical level but also involves the analysis, evaluation, summary, and transformation of relevant evidence. Ultimately, this enhances the evidence-based nursing ability of clinical nurses and the implementation climate. It is notable that only 9.68% of nurses indicated that their units did not undertake evidence-based nursing practice project(s), while 33.97% of nurses lacked EBP-related experience. In other words, a considerable number of nurses did not participate in the evidence-based nursing practice project implemented by their respective units. Nursing managers should provide an optimized EBP implementation climate for clinical nurses and create a culture that supports evidence-based nursing practice and research utilization to enhance their participation and evidence-based nursing ability [18, 20]. For example, readiness for evidence-based care, including nurses’ ability and willingness, and background analysis, should be assessed before developing targeted implementation steps. In addition, whether to participate in specific training or courses related to evidence-based nursing practice had a significant impact on the ICS score. Engaging in various types of evidence-based training can enhance the EBP knowledge, skills, competencies, and attitudes (beliefs) of nursing staff, fostering the development of EBP projects and the application of best evidence in clinical practice. Specifically, integrating theory and practice in training can more effectively attain the desired objectives, ensuring the sustainability of these attributes over time [21, 22].

In summary, developing and making available a measure of the strategic climate for EBP implementation is important, especially given recent theoretical and empirical emphasis on the organizational context’s importance for the success of EBP implementation and sustainment. If employees perceive that the organization expects, rewards, and supports the adoption and use of EBPs, they are more likely to be motivated and committed to implementing the innovation. This, in turn, can improve the probability of successful EBP implementation and sustained use. Various strategies, such as providing training and resources for employees, establishing clear expectations and performance metrics, involving employees in decision-making processes, and offering ongoing support and feedback [7, 23–25], can foster an effective implementation climate. In this study, the factors hindering the implementation climate were primarily related to resource support (e.g., financial, training and support structures). Leaders must provide resources to build EBP competency, support EBP work, give support, and navigate barriers alongside clinical nurses. Simultaneously, considering the cultural characteristics of different implementation contexts, it is essential to analyze hindering and facilitating factors individually. Strategies should be formulated to connect evidence to action in various aspects, promoting evidence transformation, implementation, and sustention in the local climate.

## Limitations

The sample size was relatively small, and only two hospitals in the central region were investigated. Additionally, due to the absence of reports on the application of the ICS in China, only the ICS in the Chinese mainland and the reliability and validity test were studied in the previous studies. Unfortunately, we did not include more related scales in this study to assess their reliability and validity in this population. Considering the geographical limitations of our study subjects, future research can conduct multi-center studies to increase the sample size, offering more comprehensive insights and reference points for a deeper understanding of the implementation climate of evidence-based nursing practice in China and its influencing factors.

## Conclusion

This study indicates that clinical nurses surveyed in central China hospitals exhibit a high level of awareness regarding the organizational atmosphere for organizing evidence-based nursing practice. However, we fonund that nurses who expressed satisfaction with their current income, engaged in evidence-based nursing practice within their units or individually, and underwet relevant training in evidence-based knowledge demonstrated a stronger sense of organizational implementation climate identity. Consequently, organizational managers should proactively offer comprehensive resource support to enhance the implementation climate of evidence-based nursing practice.

## Relevance to clinical practice

The results of this study underscore the need for organizational attention to employees’ perceptions of the significance of implementing innovation within the organization. This involves consistently addressing nurses’ expectations, rewards, and support for the organization in the application and implementation of EBP. This can be achieved by actively enhancing key internal factors influencing EBP implementation (i.e., the organizational implementation environment) [8], such as resources and policy support (performance incentives, training policies, etc.). Consequently, this can further enhance the implementation and compliance of clinical nurses with EBP [26, 27], provide them with a more positive implementation attitude [17, 28], and perpetuate a positive incentive.

### Electronic supplementary material

Below is the link to the electronic supplementary material.

Supplementary Material 1

## Data Availability

The data used in the study are available from the corresponding author upon reasonable request.
